# Identification of a Rare *PSEN1* Mutation (Thr119Ile) in Late-Onset Alzheimer’s Disease With Early Presentation of Behavioral Disturbance

**DOI:** 10.3389/fpsyt.2020.00347

**Published:** 2020-05-14

**Authors:** Shouzi Zhang, Xiang Li, Li Zhang, Xiangyan Meng, Li Ma, Guangze Zhang, Haiyan Wu, Ling Liang, Meng Cao, Fan Mei

**Affiliations:** ^1^ Psychiatry Department, Beijing Geriatric Hospital, Beijing, China; ^2^ Institute of Systems Biomedicine, Department of Pathology, School of Basic Medical Sciences, Peking University Health Science Center, Beijing, China

**Keywords:** Alzheimer’s disease, behavioral disturbance, *PSEN1* mutation, whole genome sequencing, rare coding variants

## Abstract

Alzheimer’s disease (AD) is the most common form of neurodegenerative dementia. In this study, whole genome sequencing identifies one rare and likely pathogenic mutation in the *presenilin 1 (PSEN1)* gene (c.356C > T, p.T119I) associated with a frontal variant of AD. Affected individuals in the kindred developed late-onset cognitive decline accompanied with early presentation of psychiatric symptoms. Positive amyloid PiB PET tracing suggested presence of pathophysiological biomarker for AD. Whole genome sequencing analysis evaluated rare coding mutations in susceptible genes for various types of dementia and supported the role of *PSEN1* as a causal gene. Identification of this T119I variant in *PSEN1* might broaden the spectrum of genetic basis and clinical diversity of familial AD.

## Introduction

Cognitive deficits resulting in compromised social and occupational function are typical features of dementia. Alzheimer’s disease (AD) is the most common form of dementia (1). The presence of neuritic plaques and neurofibrillary tangles is the primary neuropathological hallmark for AD (2). Positive cerebrospinal fluid (CSF) or positron emission tomography (PET) markers for cerebral Aβ and tau deposition provide supportive evidence for AD pathology (3). Typical AD presents insidious memory decline. However, an estimated 6%–14% of AD cases exhibit different clinical features from the typical amnestic form (4, 5). The most common AD variants include posterior cortical atrophy, logopenic aphasia and the frontal variant (6). Posterior cortical atrophy is characterized by visuospatial agnosia and visual disorientation (7). Clinical features of logopenic aphasia include prolonged word-finding and impaired auditory verbal short-term memory (8). The frontal variant of AD mainly presents as impairments of behavior and executive function, and overlaps with psychiatric disorders and a behavioral variant of frontotemporal dementia (9). In addition, atypical features of spastic paraparesis, early Parkinsonism, seizures, and cerebellar signs have been reported in patients with familial AD (10). Analysis of genetic factors underpinning the phenotypic diversity may provide implications for AD diagnosis and mechanism.

In 1%–2% of AD patients, the disease is inherited in an autosomal dominant pattern (2). Mutations in three genes *presenilin 1 (PSEN1)*, *presenilin 2 (PSEN2),* and *amyloid precursor protein (APP)* account for the highly penetrance of AD inherited in the mendelian form. *PSEN1* mutations constitute the majority of familial AD cases, and more than 300 mutations in *PSEN1* have been reported (10). The most frequently used approach for the identification of pathogenic variants in autosomal dominant inherited AD is Sanger sequencing of the coding regions in these three causal genes. However, this method is unable to judge the contribution of other disease-related genetic factors to the phenotypic diversity. In this study, we comprehensively analyzed susceptible genes for various types of dementia through whole genome sequencing and confirmed the mutation in *PSEN1* (c.356C > T, p.T119I) as the primary causal variant for a non-amnestic AD.

## Materials and Methods

### Subjects and Samples

Three affected individuals in the index family were available for clinical assessment, and two of them took genetic tests. This study was approved by the ethics committee of Beijing Geriatric Hospital. Informed consent was provided by each subject before sample collection according to the Helsinki Declaration. Neurological examination, neuropsychological assessment, neuroimaging, and laboratory blood tests were performed.

### Whole Genome Sequencing and Analysis of Candidate Genes

Genomic DNA was extracted from blood samples by standard protocols using the QIAamp DNA Mini Kit (QIAGEN, Cat# 51104). Whole genome sequencing was carried out on genomic DNA employing the strategy of 2x100 paired-end with Novogene using HiSeq X10 (Illumina). Sequence reads were aligned to the human genome using the Burrows–Wheeler alignment (BWA) algorithm 0.7.10-r789 (11). Genome Analysis Toolkit 330 (12), SAMtools 1.1 (13), and Picard Tools are employed for downstream processing. Single nucleotide variants and indels were subsequently called with the SAMtools suite (mpileup, bcftools, vcfutil). Variants were annotated by ANNOVAR software pipeline (14) based on the Ensembl database (release 67).

### Sanger Sequencing

The primers listed below were used for PCR. *PSEN1* exon 5 forward-TGTTGGAGGTGGTAATGTG, reverse- TAGATCAGTTAAGTTACTGTGACAAG. The products of amplification were subjected to Sanger sequencing and results were analyzed with Vector NTI and Chromas Lite software.

### Apolipoprotein E Genotyping

APOE genotyping was carried out as previously reported (15). Genomic DNA was amplified, using primers as follows: forward-TCCAAGGAGCTGCAGGCGGCGCA, reverse-ACAGAATTCGCCCCGGCCTGGTACACTGCCA.

Amplification product was then digested with five Hha1 for 2 h at 37°C and subjected to electrophoresis. The genotypes were determined by the size of DNA fragment.

## Results

### Clinical Characteristics of a Late-Onset AD Pedigree With Early Presentation of Psychiatric Symptoms

**Figure 1:**
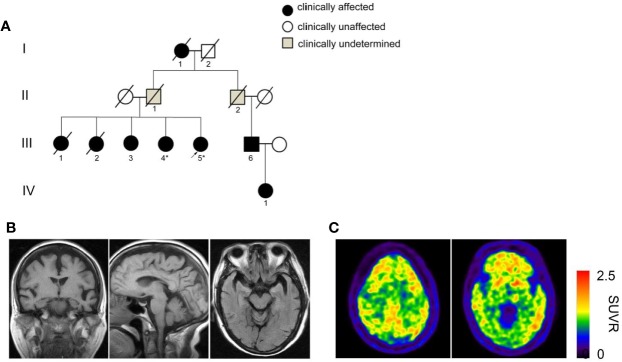
Pedigree with dementia and clinical assessment. **(A)** A four-generation pedigree with late-onset dementia and early presentation of behavioral disturbances. The proband is indicated by an arrow. Squares denote male, circles denote female. Slashes represent deceased family members. Black solid symbols indicate affected individuals with dementia, white symbols suggest unaffected individuals and gray symbols indicate clinically undetermined cases. The deceased individuals I-1, III-1 and III-2 were presumed to have been affected based on the medical history provided by relatives. Individuals whose DNA has been sequenced are shown with an asterisk. **(B)** Cranial MRI images of the proband III-5 indicate atrophy of the temporal, frontal cortex and mild shrinkage of hippocampus. **(C)** ^11^C-PiB PET shows diffuse amyloid retention in bilateral frontal, parietal and temporal cortex.

A four-generation dementia pedigree with eight affected individuals was shown in **Figure 1A** and three living individuals (III-3, III-4, and III-5) were available for clinical evaluation. The proband III-5 is a 76-year-old right-handed woman with 11 years of education. She first came to memory clinic due to cognitive decline and behavioral disturbances. At 68 years, this patient began to show executive dysfunction and recent memory impairment. She was unable to operate simple implements and do housework in daily life. Her competency for financial management was largely compromised. Behavioral disturbance and personality changes were remarkable, including apathy, social withdrawal, verbal aggression, and irritability. A diagnosis of depression and dementia was made. In the following years, amnestic and behavioral symptoms progressed over time. She developed a sudden worsening of memory at age of 72, accompanied with visual hallucination and persecutory delusions, and was partially incapable of self-care. Evaluation of neurologic symptoms was negative, including myoclonus, seizure, cerebellar syndrome, ataxia, spastic paraparesis, and extrapyramidal signs. The laboratory tests were normal in serum folate, HIV, vitamin B12, thyroid function, and syphilis serology. There was no history of central nervous system infection, head injury, stroke, transient ischemic attack, or heart disease.

Neuropsychological assessment at 5 years after onset of initial symptoms showed scores on the Mini-Mental-State Examination (MMSE) of 16, the Montreal Cognitive Assessment (MoCA) of 12, the Clinical Dementia Rating (CDR) of 2, and the neuropsychological inventory (NPI) of 27. Naming (by Boston naming test) was intact, whereas phonemic verbal fluency was slightly declined (11 words in 1 min) and semantic fluency (9 words in 1 min) was significantly impaired. She had difficulty in word list recall and recognition. In her second visit to our clinic upon 8 years after the onset of symptoms, her cognitive impairment and psycho-behavioral symptoms had been worsened with scores on the MMSE of 2, MoCA of 1, CDR of 3, and NPI of 32 (**Table 1**, year 2016 and 2019).

**Table 1:** Neuropsychological tests of the proband III-5 in year 2016 and 2019.

Cognitive Function	Test	Score (2016)	Score (2019)
Dementia	MMSE MoCA CDR	16 12 2	2 1 3
Language	BNT	20	3
BPSD	NPI	27	32
Activities of Daily Living	Barthel Index	85	70

Brain MRI revealed atrophy of the frontal and temporal lobe as well as mild shrinkage of hippocampus (**Figure 1B**). To make clinical differential diagnosis from the behavioral variant of frontotemporal dementia, ^11^C-labled Pittsburgh B (PiB) positron emission tomography/computed tomography (PET/CT) scans of the proband was performed. Qualitative reading revealed profound bilateral uptake of amyloid tracer in frontal, parietal and temporal cortex (**Figure 1C**). These results supported a diagnosis of AD based on the IWG-2 diagnostic criteria (3). The oral drug regimen included memantine and quetiapine with poor response.

The proband’s grandmother I-1 had dementia and died at age of 83. The proband’s biological father II-1 died in his 60s with no clear sign of dementia and her mother died of accidents at approximately 75 years old. The older sisters III-1 presented with memory decline and wandered off in her 60s. She worsened rapidly and died of pneumonia in her 70s (**Table 2**). The older sister III-2 showed memory decline and hallucination at age of 73. She became bedridden after 5 years of symptom onset and did not know her family. She died at age of 80. Affected individual III-3 began to show memory decline and delusion at 70 years old. She was not able to find home and express herself well. Eight years after symptoms onset, she became bedridden and always screamed at night during her living in a nursing home. The proband’s sister III-4 developed cognitive and psycho behavioral symptoms at age of 74 years old. She became bedridden after a gall-bladder surgery in 2016 and fed by gastric tube. The proband and the affected individual III-4 are not carriers of the risk APOE ε4 allele (**Table 2**).

**Table 2:** Onset age and disease duration of the affected individuals in the kindred.

Patient No.	Gender	Onset Age (Years)	Duration (Years)	APOE
III-1	Female	~60s	10	–
III-2	Female	73	7	–
III-3	Female	70	15	–
III-4	Female	74	8	ε3/3
III-5	Female	68	8	ε3/3

### Whole Genome Sequencing Analysis Reveals a Rare Missense Mutation in *PSEN1*

**Figure 2:**
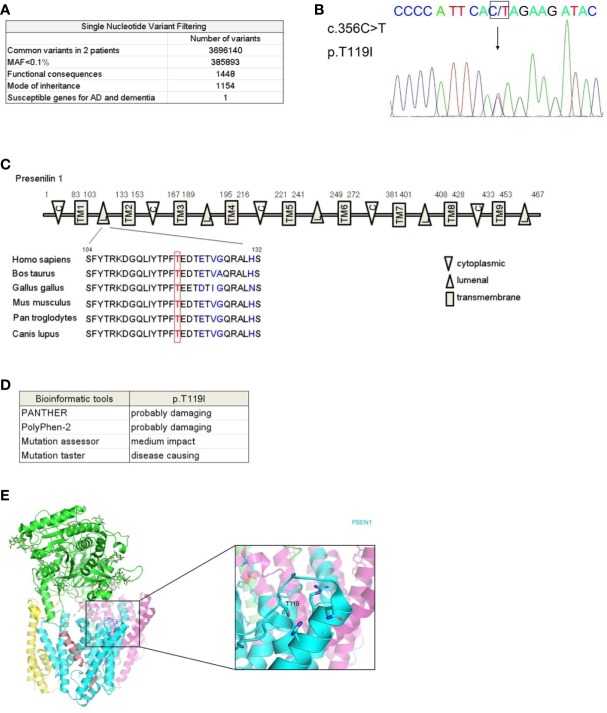
Identification of a rare mutation in *PSEN1* associated with dementia. **(A)** Workflow for the whole genome sequencing analysis of the affected individuals III-4 and III-5. MAF, minor allele frequency. **(B)** Sequence chromatograms showing heterozygous c.356C > T mutation in *PSEN1* in affected individuals. DNA sequence with this mutation was shown, with an arrow indicating the site of mutation. **(C)** Schematic illustration of presenilin 1 protein domains. C stands for cytoplasmic loop, TM stands for transmembrane domain, L stands for luminal loop. Alignment of luminal loop spanning 104-132 amino acids across species is shown. **(D)** Prediction of functional impact of PSEN1 T119I mutant by published algorithm. PANTHER (http://www.pantherdb.org/tools/csnpScoreForm.jsp), PolyPhen-2 (http://genetics.bwh.harvard.edu/pph2), Mutation assessor (http://mutationassessor.org), and Mutation taster (http://www.mutationtaster.org). **(E)** The location of the mutated residue T119 in the hydrophilic loop of human PSEN1 protein. Shown is the crystal structure of the human gamma-secretase complex with PSEN1 protein in cyan. The image was based on the structure from PDB 6IYC and generated using PyMOL.

To determine the genetic basis for this AD kindred, whole genome sequencing was performed on genomic DNA collected from the affected individuals III-4 and III-5. We analyzed the sequencing data using the GATK pipeline (16) and obtained 3696140 variants that were present in both patients (**Figure 2A**). We excluded variants with minor allele frequency above 0.1% (17) in multiple databases including the 1,000 Genome Project, the Single Nucleotide Polymorphism database (dbSNP 129), the NHLBI-GO Exome Sequencing Project ESP6500 and the East-Asia population of the Exome Aggregation Consortium (ExAC) database and 385,893 variants were left. After filtering for variant with functional consequences (missense, nonsense, splice variants, and indels), 1,448 variants remained and 1,154 out of these variants were in consistence with dominant mode of inheritance. We next compared these variants with 106 susceptible genes for AD, AD-related pathological traits and other dementia (18) (**Supplementary Table 1**) and found only one missense mutation in *PSEN1* (c.356C > T, p.T119I) was carried by the affected individuals. This variant in *PSEN1* was not reported in any SNP database. Sanger sequencing validated the missense mutation in the affected individuals (**Figure 2B**). We further employed a family-based approach to investigate the probabilities of sharing by multiple affected relatives (19), and statistical analysis achieved significance (*P*= 5.45× 10^−4^). To exclude any possibility of structure variation, we assessed *APP* duplication by Control-FREEC algorithm (20) and found the affected individuals III-4 and III-5 bearing no copy number variation in the loci.

This mutation in *PSEN1* (c.356C > T) is located on the exon 5 and the amino acid T119 is evolutionarily highly conserved across species (**Figure 2C**). The likely pathogenicity was predicted by published algorithms and these predictive analyses of functional impact reveal the substitution of threonine to isoleucine is probably damaging (**Figure 2D**). From the perspective of protein structure, T119 is within the first hydrophilic loop. However, conversion of hydrophilic threonine to hydrophobic isoleucine may lead to a change in protein conformation (21) (**Figure 2E**).

## Discussion

In this study, we report a rare missense *PSEN1* mutation (c.356C > T, p.T119I) in a late-onset AD pedigree with early presentation of behavioral disturbance. We performed whole genome sequencing and proved that *PSEN1* but not other susceptible genes for dementia contributing to the genetic cause of the frontal variant of AD.

The kindred presented with an atypical clinical presentation, however, positive PiB-PET tracing supports a diagnosis of AD. Specifically, the index patient developed progressive apathy, behavioral disinhibition, stereotyped behaviors, and executive dysfunction along with cognitive decline. Neuropsychological assessments revealed greater impairments in executive domain than that in the memory domain. Whole genome sequencing analysis excludes polygenic risk from rare single nucleotide variations in known susceptible genes for frontotemporal dementia, cerebral amyloid angiopathy, hippocampal sclerosis, Creutzfeldt-Jakob disease, Gerstmann-Straussler syndrome, cerebral autosomal dominant arteriopathy with subcortical infarcts, and leukoencephalopathy, or structural variation of *APP*, but supports a causal role of a rare missense mutation in *PSEN1*. Despite the fact that a majority of affected individuals bearing *PSEN1* mutations initially affected with amnestic AD, atypical presentations including behavioral change, language impairment, dyscalculia, and dysexecutive syndrome were also identified (10). Several *PSEN1* mutants (p.I83T, p.L113P, p.M139V, p.M139T, p.W165C, p.L166R, p.E184G, p.P264L, p.R269H, p.R278I, p.E280G, p. [S290C; T291_S319del], p.P355S) have been associated with non-amnestic AD (10, 22–26). Moreover, carriers of *PSEN1* mutations could develop frontotemporal dementia, dementia with Lewy bodies, Pick’s disease, amyotrophic lateral sclerosis, Parkinsonism, spastic paraparesis, cerebral amyloid angiopathy, and corticobasal syndrome (**Supplementary Table 2**, according to the data from the website www.alzforum.org). These data support the notion that each association of the genetic determinant with its specific phenotypic manifestation is pivotal. Our study suggested a rare missense mutant p.T119I links to the frontal variant of AD.

It has been suggested that pathogenic *PSEN1* mutations are closely associated with early onset AD. Specifically, *PSEN1* mutations within the first hydrophilic loop correlate with a much younger age of symptom onset (10). For example, carriers of Y115C, Y115H, T116N, E120K, and S132A variants presented mean age at onset of 39, 34, 34, 35, 59 years old, respectively. Structure analysis that mapping AD-associated mutations at the PS1-APP interface indicates that these residues in the first hydrophilic loop likely mediate substrate recruitment and delivery to the active site (21). It lends strong support that functional impact of this region on the pathogenic process of AD is influential. In our study, carriers of T119I variant in this pedigree uniformly presented with late-onset AD. This phenotype might be derived from unknown protective genetic factors that delayed the age of symptom onset. For example, homozygous APOE3 R136S mutation has been suggested to largely postpone the expected age of clinical onset in the *PSEN1* E280A mutation carrier (27).

One major clinical feature of this PSEN1 T119I pedigree is the early presentation of behavioral change. Disturbance in neuroplasticity and functional alterations in circuitry activity underlie mechanisms of psychiatric symptoms (28–31). It is noteworthy that in addition to being an integral component of ϒ-secretase that responsible for the intramembranous cleavage of both APP and Notch, presenilin 1 regulates long-term potentiation, neurogenesis, homeostatic scaling, neurotransmission and calcium homeostasis (32–37). It thus suggests a molecular basis for the onset of psychiatric symptoms owing to *PSEN1* mutations.

Interestingly, PSEN1 T119I variant has recently been reported in one Argentine family and one Korean sporadic AD case (38, 39). To our knowledge, the pedigree we reported is the first Chinese kindred with the T119I mutation. The age of onset varies from 49 to 71 years old in the Argentine family in which some affected individuals carry the APOE ε4 allele, whereas the sporadic Korean patient has an age of onset at 64 years old. The age of onset in this Chinese pedigree is relatively late comparable to the previous reports. The major clinical symptom of the previously reported T119I carriers is memory loss, while our study characterizes symptoms associated with psychiatric disorders. However, the proband in the Argentine family also developed depression after a life event. Thus, our report have broadened phenotypic diversity of *PSEN1* T119I carriers.

One limitation of this study is that upon the clinical assessment, the proband’s older sisters had already been at a stage of severe dementia with marked functional disability. It thus makes impossible to evaluate detailed behavioral changes, and we only have description of medical history provided by family members. In addition, lack of autopsy data from this PSEN1 T119I pedigree makes characterization of the pathological features of this frontal variant difficult. Although positive PiB/PET amyloid tracing supports AD pathology, previous studies suggest that the presence of non-AD pathology might contribute to the atypical presentation of a cortical phenotype (40). Moreover, biological experiments to examine a functional change of T119I mutant in amyloid production and neuroplasticity are warranted.

In summary, we have identified a rare *PSEN1* mutation (c.356C > T, p.T119I) associated with an atypical presentation of psychiatric symptoms in AD. This variant is located in the first hydrophilic loop and predicted to be disease causing. Whole genome sequencing excludes the presence of rare mutations in other known susceptible genes for dementia and supports the role of *PSEN1* as a causal gene in this pedigree. This study broadens the spectrum of genetic basis and clinical diversity of *PSEN1* carriers with AD.

## Data Availability Statement

The sequencing data in our manuscript has been deposited in the National Center for Biotechnology Information (NCBI) Sequence Read Archive (SRA) under the Bioproject PRJNA615967.

## Ethics Statement

This study was approved by the ethics committee of Beijing Geriatric Hospital. Written informed consent including the publication was provided by each subject before sample collection according to the Declaration of Helsinki.

## Author Contributions

FM and SZ conceived the study and designed the major experiments. XL and GZ performed analysis of whole genome sequencing. FM, SZ, LZ, LM, HW, LL, and MC performed experiments. XM performed Sanger sequencing analysis. The manuscript was written by FM and SZ.

## Funding

This study was supported by grants including the National Key Research and Development Program of China Grant (2016YFA0500302); National Natural Scientific Foundation of China (31420103905, 81430056, 81372491, and 81200829); Beijing Natural Science Foundation Key Grant (7161007); and the Lam Chung Nin Foundation for Systems Biomedicine.

## Conflict of Interest

The authors declare that the research was conducted in the absence of any commercial or financial relationships that could be construed as a potential conflict of interest.
