# Supplementary material for: Identification of a Rare PSEN1 Mutation (Thr119Ile) in Late-Onset Alzheimer’s Disease With Early Presentation of Behavioral Disturbance
Source: Front Psychiatry. 2020 May 14;11:347. doi: 10.3389/fpsyt.2020.00347 (PMC7240292; doi:10.3389/fpsyt.2020.00347)
Supplement: Supplementary file 1 [file DataSheet_1.pdf]

AD		NP
SORL1	C1QTNF4	ABCG1
PICALM	CASP8	GALNT7
LRP1	CELF1	**NFT+CAA**
A2M	COBL	ADI1
ABCA1	DSG2	HDAC9
CLU/APOJ	FBXL7	TRAPPC12
BIN1	FRMD4A	**FTD**
RAGE	GLIS3	CHCHD10
Aquaporin	HBEGF	CHMP2B
ABCA7	IGHV1-67	CSF1R
EPHA1	KANSL1	FUS
MS4A4A	KCNMB2	GRN
MS4A6E	MAPT	TARDBP
INPP5D	MEF2C	VCP
HLA-DRB1	MVB12B	**NP+NFT**
HLA-DRB5	NME8	ECRG4
CR1	OSTN	**Leukodystrophy**
CD33	PFDN1	EIF2B1
TREM2/TYROBP	PILRA	EIF2B2
CD2AP	PLCG2	EIF2B3
PTK2B	PLD3	EIF2B4
NYAP1	PLXNA4	EIF2B5
ECHDC3	PPP2CB	LMNB1
SPI1	RIN3	**FBD/FDD**
SLC24A4	SLC10A2	ITM2B
FERMT2	SLC24A4	**HS**
ADAM10	SLC2A4A	LMX1B
IQCK	SORCS1	**HPV**
WWOX	SORCS2	MTUS1
ACE	SORCS3	PDGFRL
CASS4	SORL1	**CADASIL**
ADAMTS1	TM2D3	NOTCH3
APP	TP53INP1	**CSF Tau**
PSEN1	TPBG	PLD4
PSEN2	TREML2	SRRM4
ABI3	TRIP4	**CJD/GSS**
AKAP9	UNC5C	PRNP
APOE	USP6NL	**LMdT**
BZRAP1	ZCWPW1	ZNF804B

Supplementary Table 1. Causal genes and risk factors for various types of dementia and AD-associated traits [1]. AD, Alzheimer's disease; NP, neurite plaque; NFT, neurofibrillary tangle; FTD, frontotemporal dementia; FBD, familial British dementia; FDD, familial Danish dementia; HS, hippocampal sclerosis; HPV, hippocampal volume; CADASIL, cerebral autosomal dominant arteriopathy with subcortical infarcts and leukoencephalopathy; CJD, Creutzfeldt-Jakob disease; GSS, Gerstmann-Straussler syndrome; LMdt, logical memory-delayed recall.

PSEN1 mutations	Clinical phenotypes
Q15H	Frontotemporal dementia
D40del	Frontotemporal dementia ; Alzheimer's disease
I83-M84del	Spastic paraparesis; Alzheimer's disease
M84V	Spastic paraparesis; Alzheimer's disease
L85P	Myoclonus; Spastic paraparesis; Parkinsonism; Alzheimer's disease; Corticobasal syndrome
L113P	Frontotemporal dementia
P117A	Ataxia; Alzheimer's disease
E120K	Spastic paraparesis; Parkinsonism; Alzheimer's disease
T122A	Frontotemporal dementia
S132A	Dementia with Lewy bodies; Myoclonus
A137T	Frontotemporal dementia
I143T	Myoclonus; Alzheimer's disease
M146L	Pick's disease; Alzheimer's disease
M146V	Frontotemporal dementia; Alzheimer's disease
T147P	Ataxia; Alzheimer's disease
Y154N	Spastic paraparesis; Alzheimer's disease
Y156F; Y156-R157insIY	Spastic paraparesis; Alzheimer's disease
Y159C	Parkinsonism; Alzheimer's disease
H163R	Myoclonus; Alzheimer's disease
L166P	Spastic paraparesis; Alzheimer's disease
S169P	Myoclonic seizure; Alzheimer's disease
F175del	Myoclonic seizure; Alzheimer's disease
G183V	Pick's disease
W203C	Amyotrophic lateral sclerosis
G217D	Parkinsonism; Alzheimer's diseae
Q223R	Spastic paraparesis; Alzheimer's disease
L226F	Frontotemporal dementia; Alzheimer's disease
L235P	Myoclonus; Alzheimer's disease
F237I	Spastic paraparesis; Alzheimer's disease
P242Lfs	Familial Acne Inversa
I249L	Amyotrophic lateral sclerosis; Alzheimer's disease
L250V	Myoclonus; Alzheimer's disease
V261F	Spastic paraparesis; Alzheimer's disease
V261L	Spastic paraparesis; Alzheimer's disease
L262V	Frontotemporal dementia; Alzheimer's disease
P264L	Spastic paraparesis; Alzheimer's disease; Progressive nonfluent aphasia
G266S	Spastic paraparesis; Alzheimer's disease; Cerebral amyloid angiopathy
R269G	Myoclonus; Alzheimer's disease
R269H	Myoclonus; Alzheimer's disease
V272A	Parkinsonism; Alzheimer's diseae; Subcortical dementia
V272D	Logopenia; Disorientation; Apraxia
R278I	Alzheimer's disease; Progressive nonfluent aphasia
R278K	Spastic paraparesis; Alzheimer's disease
R278S	Spastic paraparesis; Alzheimer's disease
R278T	Spastic paraparesis; Alzheimer's disease
E280G	Spastic paraparesis; Alzheimer's disease
L282V	Cerebral amyloid angiopathy; Alzheimer's disease
F283L	Alzheimer's disease; Corticobasal syndrome
P284L	Spastic paraparesis; Alzheimer's disease
P284S	Spastic paraparesis; Alzheimer's disease
L286P	Cerebral amyloid angiopathy; Alzheimer's disease
T291P	Spastic paraparesis; Alzheimer's disease
P303L	Frontotemporal dementia
P355S	Frontal variant of Alzheimer's disease
D333G	Dilated Cardiomyopathy
R352-S353insR	Frontotemporal dementia
G378E	Cerebral amyloid angiopathy; Alzheimer's disease
L381V	Spastic paraparesis; Alzheimer's disease
Y389H	Parkinsonism; Alzheimer's disease
V412I	Frontotemporal dementia
G417A	Parkinsonism; Alzheimer's disease
L424R	Spastic paraparesis; Alzheimer's disease
A434T	Parkinsonism; Alzheimer's disease
P436Q	Spastic paraparesis; Alzheimer's disease
T440del	Dementia with Lewy bodies; Alzheimer's disease
869-2A>G	Behavioral variant of frontotemporal dementia
869-22/869-23ins18	Spastic paraparesis; Alzheimer's disease
S290C; T291-S319	Spastic paraparesis; Alzheimer's disease
S290C; T291-S311delG>T	Spastic paraparesis; Alzheimer's disease
S290W, S291-R377del	Spastic paraparesis; Alzheimer's disease

Supplementary Table 2. *PSEN1* mutations associated with atypical manifestation. (Data from the website www.alzforum.org).

Reference

[1] D. Patel, J. Mez, B.N. Vardarajan, L. Staley, J. Chung, X. Zhang, J.J. Farrell, M.J. Rynkiewicz, L.A. Cannon-Albright, C.C. Teerlink, J. Stevens, C. Corcoran, J.D. Gonzalez Murcia, O.L. Lopez, R. Mayeux, J.L. Haines, M.A. Pericak-Vance, G. Schellenberg, J.S.K. Kauwe, K.L. Lunetta, and L.A. Farrer, Association of Rare Coding Mutations With Alzheimer Disease and Other Dementias Among Adults of European Ancestry. JAMA Netw Open 2 (2019) e191350.
